# Complications in ankle arthroscopy

**DOI:** 10.1007/s00167-012-2063-x

**Published:** 2012-06-05

**Authors:** Maartje Zengerink, C. Niek van Dijk

**Affiliations:** Department of Orthopaedic Surgery, Academic Medical Centre, University of Amsterdam, PO Box 22700, 1100 DD Amsterdam, The Netherlands

**Keywords:** Ankle arthroscopy, Non-invasive distraction, Complications

## Abstract

**Purpose:**

To determine the complication rate for ankle arthroscopy.

**Methods:**

A review of a consecutive series of patients undergoing ankle arthroscopy in our hospital between 1987 and 2006 was undertaken. Anterior ankle arthroscopy was performed by means of a 2-portal dorsiflexion method with intermittent soft tissue distraction. Posterior ankle arthroscopy was performed by means of a two-portal hindfoot approach. Complications were registered in a prospective national registration system. Apart from this complication registry, patient records, outpatient charts and operative reports were reviewed. Patients with a complication were asked to visit our hospital for clinical examination and assessment of permanent damage and persisting complaints.

**Results:**

An overall complication rate of 3.5 % in 1,305 procedures was found. Neurological complications (1.9 %) were related to portal placement. Age was a significant risk factor for the occurrence of complications. Most complications were transient and resolved within 6 months. Complications did not lead to functional limitations. Residual complaints did not influence daily activities.

**Conclusions:**

Our complication rate is less than half of what has been reported in literature (3.5 vs 10.3 %). The use of the dorsiflexion method for anterior ankle arthroscopy can prevent a significant number of complications. Posterior ankle arthroscopy by means of a two-portal hindfoot approach is a safe procedure with a complication rate that compares favourably to that of anterior ankle arthroscopy.

**Level of evidence:**

Retrospective prognostic study, Level II.

## Introduction

Arthroscopy of the ankle joint has become an important therapeutic tool for the management of post-traumatic and chronic ankle problems. Although the anatomy of the ankle is rather complex, ankle arthroscopy is generally perceived as ‘no problem surgery’. When compared to arthrotomy, ankle arthroscopy is related to decreased morbidity and rapid rehabilitation. For treatment of posterior ankle problems, a 2-portal hindfoot approach was developed and published [40]. Several publications have shown the safety of the portals [38, 40].

### What is known

The average complication rate in ankle arthroscopy is 10.3 % (Table 1). This rate used to be even higher, but over the last 25 years, remarkable progress has been made concerning the field of minimal invasive foot and ankle surgery. Up to 1984, the ankle joint was found difficult to approach by means of the arthroscope. Burman even found the ankle joint unsuitable for arthroscopy [6]. The percentage of complications was high: Sprague et al. [34] mentioned 24.6 % complications in the pre-distraction era. In 1984, routine (invasive) distraction of the ankle joint was advocated by Guhl, Martin et al., Barber et al. and others. The percentage of complications dropped to 13.6 % [34]. With the aim to further diminish the percentage of complications, the approach gradually changed from invasive distraction towards continuous soft tissue distraction. Recent studies reported complication rates ranging from 6.8 to 20 % [1, 11, 13, 14, 17, 27, 37, 45]. Most older studies report the complication rate of anterior ankle arthroscopy only. More recent studies report on the rate for anterior and hindfoot endoscopy [11], or for hindfoot endoscopy alone [17, 27]. Altogether, the average published rate of 10.3 % still indicates a significant risk of complication in ankle arthroscopy.

**Table 1:** Summarized literature on complications in ankle arthroscopy

Author	Year	Journal	No. of patients	No. of complications	%	Neurol.	Deep infection	Superf. Infection	CRPS	Instr. break.	Sinus tract/delayed wound healing	Nerves involved
Guhl	1986	Orthopedics	62	5	8	1	–	1	–	–	3	Unknown
Guhl	1988	Arthroscopy	131	13	10	4	–	2	–	2	–	Sural n. 3 Neuroma 1
Sprague	1989	Book	201	35	17.4	5		3	1	1	4	Sural n. 3 Saphenous n.1 Neuroma 1
Martin et al.	1989	Am. J. Sports Med.	58	9	15	5	2	2	–	–	–	Unknown
Barber et al.	1990	Foot and Ankle	53	9	17	3	1	2	1	–	2	Superf. peroneal n. 3
Ferkel et al.	1996	J. Arthroscopic Rel. Surg.	612	55	9	27	2	8	1	2	1	Superf. peroneal n. 15 Sural n. 6 Saphenous n. 5 deep peroneal n. 1
Amendola et al.	1996	J. Arthroscopic Rel. Surg.	79	6	7.6	3	–	–	–	–	3	Superf. peroneal n.1 Deep peroneal n. 2
Unger et al.	2000	Unfallchirurg	155	14	9	3	3	2	–	–	4	Superf. peroneal n. 3
Bilgin et al.	2004	Acta Orthop. Traumatol. Turc.	32	2	6	–	–	–	1	1	–	–
Galla et al.	2011	Foot Ankle Surg.	30	6	20	2	1	1	–	–	–	Sural n. 2
Deng et al.	2012	J. Foot and Ankle Surg.	251	20	8.0	9	–	8	–	–	–	Superficial peroneal n. 5 Deep peroneal n. 2 Sural n. 1 saphenous n. 1
Nickish et al.	2012	J. Bone Joint Surg.	189	16	8.5	7	1	1	2	–	–	Tibial n. 4 Sural n. 3
Total		12	1,853	190	10.3	3.7 %	0.6 %	1.6 %	0.3 %	0.3 %	0.9 %	

*No* number, *Neurol.* neurological, *Superf.* superficial, *CRPS* complex regional pain syndrome, *Instr. break.* instrument breakage, *n.* nerve

Within the spectrum of occurring complications, neurological injury has been described most. Nerve lesions and neuromas have been mentioned [1, 2, 13, 14, 16, 18, 19, 34, 36], as well as vascular injuries, false aneurysms [5, 7, 21, 23, 25, 28, 30, 31, 43], infections and synovial fistula [2, 14, 18, 19, 26, 37]. Complications related to invasive distraction are ligament injuries, pintrack infections and stress fractures [14, 34]. Furthermore, instrument breakage, complex regional pain syndrome (CRPS), compartment syndrome, thromboembolic complications and painful scars [2, 18, 37], and even iatrogenic excision of the distal fibula have been described [32].

### What is unknown and why it is important

It was recently suggested that a large number of these complications can be contributed to the continuous distraction itself [45]. If the continuous distraction set-up indeed is responsible for the high complication rate in ankle arthroscopy, then one could expect a considerably lower complication rate when using a non-distraction method. To test this hypothesis, the results in a consecutive series of patients that were treated by means of a non-fixed distraction method were analysed.

### Continuous fixed distraction versus non-fixed distraction

Continuous fixed distraction is applied by means of a distraction holder that is mounted to the operating table which is fixed to an external strap around the ankle. The amount of distraction used is maximum 50 lb of force [4]. One of the advantages is that the distraction, in combination with a 2.7-mm arthroscope, allows for a diagnostic inspection of the complete ankle joint. An eighteen-point inspection scale has been developed for this purpose [15]. In the non-distraction method, the portals are created without the foot mounted onto a device. Classically, the technique makes use of a dorsiflexed position to introduce and interchange the instruments [10]. This technique is also known as the dorsiflexion method. For treatment of posteriorly located osteochondral defects, a soft tissue distractor can be applied allowing for intermittent distraction.

Working without distraction gives better access to anterior ankle pathology because it creates an anterior working space. It, however, demands a diagnosis before the procedure is undertaken. For most joints, diagnostic arthroscopy has been abandoned. In the ankle, pre-operative workup includes CT or MRI. It has been shown that in patients in whom no definite pre-operative diagnosis was made and for whom diagnostic ankle arthroscopy was performed, only 26 % benefitted from the procedure [41]. Like in other joints, arthroscopy of the ankle is a therapeutic and not a diagnostic procedure.

### Purpose and how we answered the question

The purpose of this study was to determine the complication rate for anterior ankle arthroscopy related to the dorsiflexion method and the complications related to the 2-portal posterior ankle arthroscopy method. To gain insight into the aetiology of complications, the relationship between specific indications, surgical characteristics and the number of complications that occurred was determined.

## Materials and methods

Between July 1987 and 2006, 1305 consecutive ankle arthroscopies were performed at the University Hospital of the University of Amsterdam. The procedures were performed by 33 different orthopaedic surgeons, at a single surgery centre. Minimal follow-up was 24 months. Charts of all patients, including surgery reports, were reviewed to identify and verify all pre- or post-operative complications. In addition, it was used to identify the most recent follow-up. Patient characteristics (side, gender, age at time of surgery, whether it concerned a day care procedure, surgeon, indication, treatment and duration of surgery) were retrieved. Patients with a pre- or post-operative complication were identified. A complication was defined as ‘every event that arises as an additional problem during or following the procedure and is secondary to it’. Our hospital has an active complication surveillance system. All complications are systematically scored in a database. Apart from this active prospective scoring system, we reviewed all patient charts and looked for any uneventful post-operative course. All patients with a reported complication were asked to visit our outpatient department for a clinical follow-up examination.

At follow-up, the patients’ history was taken and a physical examination of the ankle was performed. The patient was asked for pain, limitations in activities, support requirement, limitation in walking distance and difficulties of walking on uneven surfaces. During physical examination, gait abnormality, sagittal range of motion, alignment of the foot and hindfoot and ankle-hindfoot stability were assessed. This information was used to determine the AOFAS Ankle/Hindfoot score [24].

Patients were asked how the complication had developed over time. In case of a neurological complication, usually consisting of hyposensitivity of a cutaneous part of the foot, a pin prick test was used to determine the area of altered sensation. The area was marked. For documentation, feet were photographed and nerve damage was assessed.

### Surgical technique

Both anterior and posterior ankle arthroscopy are routinely carried out as day care procedures. No prophylactic antibiotics are given. For both procedures, a 4-mm 30° angle arthroscope is used.

**Fig. 1:**
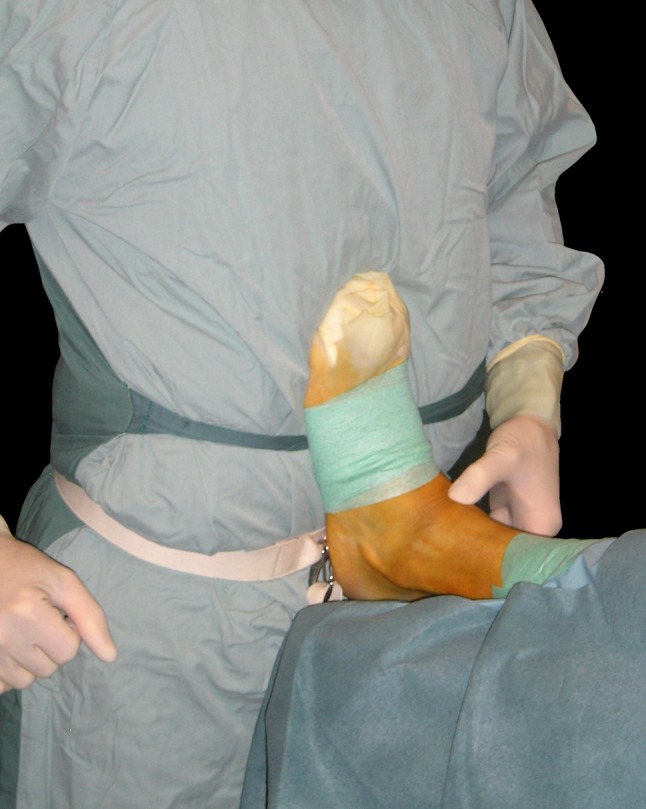
Introduction of arthroscope in fully dorsiflexed position. The foot leans against the surgeons’ belly

In anterior ankle arthroscopy, the patient lies supine with slight elevation of the ipsilateral buttock. The heel of the affected foot rests on the edge of the operating table. The surgeon can fully dorsiflex the ankle by leaning against the sole of the patients’ foot (Fig. 1). A resterilizable, non-invasive device is used for intermittent distraction [39]. The surgeon can distract the ankle joint by leaning backward. The 4-mm arthroscope is introduced in the fully dorsiflexed position, allowing complete relaxation of all surrounding structures. Under arthroscopic view, the anterolateral portal is created by introducing a spinal needle lateral to the tertiary peroneal tendon, while keeping the superficial peroneal nerve intact. Pre-operatively, the superficial peroneal nerve is identified by bringing the foot in forced plantarflexion and inversion. In case the nerve is visualized, it is important to mark its course onto the skin with a felt pen [35].

For posterior ankle arthroscopy, the patient is placed in a prone position. The same intermittent distraction device that was described for anterior ankle arthroscopy can be used when indicated. The posterolateral portal is created first. The direction of view is 30° to the lateral side. The posteromedial portal is made at the same level. After a vertical stab incision, a mosquito clamp is introduced and when scar tissue or adhesions are present, the mosquito clamp is exchanged for a 4.5 mm full radius shaver. After removal of the very thin joint capsule of the subtalar joint, the posterior compartment of the subtalar joint can be visualized. The posterior talar process can be freed of scar tissue, and the flexor hallucis longus tendon is an important landmark to prevent damage to the medial neurovascular bundle. One always stays lateral to this tendon. After removal of the thin joint capsule of the ankle joint, it can be entered, inspected and treated.

### Statistical analysis

To assess the presence of a learning curve, we selected the surgeons who had performed at least 20 operations and divided these 20 operations in a first series of 10 and a second series of 10 operations. Complication rates of the two series were compared using a Fisher’s exact test since expected counts of less than 5 were present. A *p* value of <0.05 was considered statistically significant. The influence of several other risk factors on the occurrence of complications was evaluated using multivariable logistic regression analysis with backward selection procedure. Univariate analysis was performed before entering the risk factors in the multivariable model. Student’s *t* tests were performed for continuous variables to assess differences between the complication group and non-complication group (age and duration of surgery). Chi-square tests were performed for the assessment of the difference in complication rates of categorical variables (gender, side, type of operation and whether it considered a clinical or day care procedure). We used a univariate 0.1 significance level for entering the risk factors in the multivariable analysis. Subsequently, odds ratios (OR) with accompanying 95 % confidence interval (CI) were calculated for the risk factors that significantly contributed to the logistic regression model (*p* < 0.05).

Statistical analysis was performed using SPSS 15.0 (SPSS Inc. Headquarters, 233 S. Wacker Drive, 11th floor, Chicago, Illinois 60606, USA).

## Results

Median follow-up was 7.5 years (range 2–20). Altogether, a total of 1,305 ankle arthroscopies in 1176 patients were performed. Of the 122 patients that were operated more than once, 52 were operated bilateral. Seven patients were operated three times, in three cases bilateral. Three patients were lost to follow up, as their medical charts were lost. We did, however, have their surgical files as well as their complication documentation. They did not have a registered complication. Sixty-three per cent was male and 37 female. The mean age at surgery was 33 years (SD 11.0). In 54 %, it involved the right and in 46 % the left ankle.

Nine hundred and five anterior ankle arthroscopies (69.3 %), 311 posterior (23.8 %) and 17 combined anterior and posterior arthroscopies (1.3 %) were performed. Twenty-one patients had a combined right and left ankle arthroscopy (in one session). Median duration of surgery was 37 min (range 4–195).

Forty-six complications in 43 patients were identified (Table 2). The overall complication rate was 3.5 %. The complication rate for hindfoot endoscopy alone was 2.3 %. Pulmonary embolism in a 32-year-old woman 1 week post-operatively was the most serious complication. The patient had undergone arthroscopy to determine the articular damage after sustaining an acute ruptured anterior talofibular ligament (ATFL). Because of (open) suturing of the ATFL, she received a cast for 6 weeks. She did not receive anticoagulation therapy while she was wearing the cast. The most common complication that occurred was neurological injury: 25 complications. These can be subdivided into the nerves that were injured (Table 2). Six of 7 patients with superficial infection were treated with 5 days of prophylactic antibiotics. None of them had a positive culture. In all patients, symptoms resolved within a week. Two patients with a deep infection had re-surgery and received intravenous antibiotics. One of them was a patient with sinus tract formation that did not receive prophylactic antibiotics and developed a deep infection 12 days after surgery.

**Table 2:** Patient characteristics of patients with a complication

No.	Age	Gender	Side	Day care	Dura-tion	FU	A/P	Indication	Treatment	Complication	Nerve involved	Residual complaints	Aofas
1	26	F	R	–	45	17	A	Soft tissue impingement	Removal of ant. soft tissue	Neurological	Deep peroneal n.	+	100
2	33	F	L	–	115	16	A	Ant. osteochondral defect	Ecd of lateral talar ocd	Neurological	Deep peroneal n.	+	100
3	31	F	R	–	85	16	A	Diagnostic arthroscopy	Nettoyage	Pulm. embolism	−	−	100
4	37	M	L	–	90	16	A	Nettoyage	Removal of osteophyte	Instrument breakage	−	LTFU	−
5	31	M	L	+	20	12	A	Osteophyte	Removal of osteophyte	Deep infection	−	−	88
6	40	M	L	+	35	12	A	Bony impingement	Removal of osteophyte	Superficial infection	−	−	95
7	18	M	L	+	25	12	A	Nettoyage	Removal of soft tissue + osteophyte	Sinus tract formation	−	LTFU	−
8	46	F	R	+	35	11	A	Osteophyte	Removal of osteophyte	Sinus tract formation Neurological	− Sural n.	− ? (HMSN)	85
9	32	M	R	+	?	10	A	Osteophyte	Removal of osteophyte and loose body	Neurological	Superficial peroneal n.	+	85
10	37	M	L	+	35	10	A	Bony impingement	Removal of osteophyte	Neurological	Saphenous n.	–	100
11	31	M	L	+	25	10	A	Loose body	Removal of loose body	Sinus tract formation	−	−	78
12	24	F	R	+	25	10	A	Soft tissue impingement	Removal of anterior soft tissue	Neurological	Superficial peroneal n.	+	78
13	32	M	R	+	25	10	A	Bony impingement	Removal of osteophyte + synovectomy	Sinus tract formation	−	LTFU	??
14	34	F	R	+	35	10	A	Ant. osteochondral defect	Ecd of lateral talar ocd	Neurological	Sural n.	+	72
15	55	M	R	+	45	9	A	Bony impingement	Removal of osteophyte	Neurological	Superficial peroneal n.	−	83
16	33	M	R	+	45	9	P	Posterior bony impingement	Reduction of posterior talar process + release of fl. hall. longus	Neurological	Tibial n., calc. branch	+	90
17	54	M	R	+	50	9	A	Loose body	Removal of loose body	Deep infection	−	−	80
18	38	F	L	+	30	9	A	Soft tissue impingement	Removal of soft tissue	Sinus tract formation	−	−	95
19	56	F	L	+	30	8	A	Osteochondral defect	Ecd of medial talar ocd	CRPS	−	−	94
20	29	F	R	+	88	8	C	Ant. + post. complaints	Ecd of posterior talar ocd	Neurological superficial infection	Superficial peroneal n. −	+ −	91
21	51	F	R	+	22	8	T	Tenosynovitis	Release of posterior tibial tendon	Neurological	Saphenous n.	? (degen. sp.)	71
22	35	M	L	+	37	7	P	Loose body	Removal post. calcification/ossicle	Superficial infection	−	−	73
23	27	F	R	+	30	7	A	Osteochondral defect	Ecd of medial talar ocd	Superficial infection	−	−	80
24	54	F	R	+	40	7	A	Osteochondral defect	Ecd of lateral talar ocd	Superficial infection neurological	− Superficial peroneal n.	− +	90
25	29	M	L	+	35	6	A	Bony impingement	Ecd of tibial ocd + removal osteophyte	Neurological	Deep peroneal n.	−	??
26	51	M	L	+	25	6	A	Osteochondral defect	Ecd of lateral talar ocd + removal loose body	Neurological	Superficial peroneal n.	+	98
27	32	F	R	+	23	6	P	Posterior complaints	Removal of post. soft tissue + release of fl. hall. longus	Neurological	Tibial n., calc. branch	−	75
28	42	M	R	+	20	6	A	Anterior impingement	Removal of osteophyte	Neurological	Superficial peroneal n.	−	98
29	21	F	R	+	31	6	A	Anterior impingement	Removal of soft tissue	Neurological	Superficial peroneal n.	+	100
30	48	M	R	+	15	5	A	Osteochondral defect	Ecd of medial talar ocd + removal of osteophyte	Neurological	Deep peroneal n.	+	98
31	47	F	R	+	38	5	A	Soft tissue impingement	Removal of soft tissue	Vascular damage	−	LTFU	−
32	33	M	L	+	17	5	A	Anterior impingement	Removal of osteophyte + ecd of medial talar ocd	Neurological	Sural n.	+	98
33	31	F	R	+	36	5	P	Osteochondral defect	Ecd of posterior talar ocd	Neurological	Superficial peroneal n.	+	98
34	43	M	R	+	52	5	A	Osteochondral defect	Ecd of lateral talar ocd + removal of osteophyte	Neurological	Deep peroneal n.	+	90
35	30	M	L	+	17	5	A	Anterior impingement	Removal of loose body + osteophyte	Neurological	Saphenous n.	+	97
36	42	F	L	+	54	4	A	Anterior impingement	Removal of osteophyte + nett. arthritis	Superficial infection	−	−	62
37	17	M	R	+	30	3	A	Anterior impingement	Removal of ossicle + synovectomy	Neurological	Superficial peroneal n.	LTFU	??
38	32	M	L	–	32	3	P	Posterior impingement	Removal of soft tissue + loose body deltoid ligament	Synovitis	−	−	98
39	25	M	L	+	25	2	P	Posterior impingement	Reduction of posterior talar process + release of fl. hall. longus	Sinus tract formation	−	−	100
40	27	M	R	+	30	2	P	Posterior impingement	Removal of os trigonum	Sinus tract formation	−	−	88
41	42	M	R	+	28	2	A	Anterior impingement	Removal of osteophyte	Neurological	Superficial peroneal n.	+	78
42	36	F	L	+	28	2	A	Osteochondral defect	Ecd of lateral talar ocd + removal of soft tissue	Neurological	Superficial peroneal n.	+	85
43	35	M	L	+	34	2	A	Anterior impingement	Removal of osteophyte	Superficial infection	−	−	82

*No.* number, *Age* age in years at time of surgery, Gender (*M* male, *F* female), Side (*L* left, *R* right), Day care (+ = day care surgery, − = clinical surgery), *Duration* duration of surgery in minutes, *FU* = follow-up in years, A/P = type of surgery (*A* anterior arthroscopy, *P* posterior arthroscopy, *C* combined anterior and posterior arthroscopy, *T* tendoscopy), *Indication* indication of surgery before treatment (*ant.* anterior, *post.* posterior), Treatment = final surgical treatment given (*ecd* excision, curettage and drilling, *fl. hall. longus* flexor hallucis longus tendon), Complication = type of complication that occurred (*pulm.* pulmonary, *CRPS* complex regional pain syndrome), Nerve involved (*n.* nerve, *calc. branch* calcaneal branch), Residual complaints (+ = residual complaints, − = no residual complaints, *LTFU* lost to follow-up, *HMSN* hereditary motor and sensory neuropathy, *degen. sp.* degenerative spine disease), *Aofas* AOFAS Ankle/Hindfoot score

Thirty-eight patients with a complication were seen at our hospital for re-evaluation. Five patients were lost to follow up. Among these were the patient with vascular damage and the one where instrument breakage had occurred. Both patients had a recorded uneventful recovery. Two patients with sinus tract formation and one patient with hypoesthesia in the area of the superficial peroneal nerve could not be traced. Chart review showed that the first two patients had no complaints at their last follow-up.

The 38 patients seen at follow-up accounted for 41 complications. Of the 41 complications, 22 healed without complaints, 17 had persistent complaints, and these were all of neurologic origin. Two could not be assessed because of situations not related to the arthroscopy. These situations included degenerative spine disease with loss of sensibility in both feet in one patient and the development of hereditary motor and sensory neuropathy in another patient.

Seventeen of 25 patients with a neurological complication reported persisting symptoms at follow-up (68 %). This consisted of hypoesthesia of a cutaneous part of the foot. None of these patients had functional limitations. Five patients were symptom free. One patient was lost to follow up, and two could not be assessed because of the reasons previously mentioned.

Concerning the assessment of a possible learning curve, we identified 7 surgeons that performed 20 operations or more. Complication rate in the first 10 operations was 5.7 %, and in the second 10 4.3 %. The difference was not significant. In the 13 predetermined indication groups, all complication rates were variable between 0 and 8.3 %. Univariate analysis of the risk factors revealed a significant difference for age between patients with and without complications (*p* = 0.047). Mean age of the patients without a complication was 33.3 years (SD 11.0), and of patients with a complication, this was 36.6 years (SD 9.3). Mean duration of surgery in the group without a complication was 37.3 min (SD 18.3), and in the group with a complication, this was 37.7 min (SD 19.5). The difference was not significant. Furthermore, no relationship was found between complication rate and gender, type of operation (anterior, posterior or combined surgery), whether it involved a day care procedure or not and side of the ankle. Multivariable logistic regression analysis showed that only age was a significant risk factor for the occurrence of complications with an OR of 1.03 (95 % CI, 1.00–1.05). The mean AOFAS Ankle/Hindfoot score at follow-up was 88 (SD 10).

## Discussion

The most important finding of the present study was an overall complication rate of 3.5 % for ankle arthroscopy related to the dorsiflexion method. All patients in whom a complication occurred were satisfied with the outcome of the arthroscopic procedure at long-term follow-up. None of the initial complications led to a functional limitation. Our complication rate of 3.5 % compares favourably to the average of 10.3 % found in literature. The difference is most likely even bigger as we scored our complications prospectively, instead of retrospectively like other studies [1–3, 11, 13, 14, 17–19, 27, 34, 37, 45]. Retrospective studies tend to score lower complication rates when compared to prospective documentation.

Neurological complications (1.9 %) were most common and accounted for half of the total amount of complications in our series. In a recent study, in which continuous distraction was used, a neurological complication rate of 5.4 % was found [45]. Other authors that use continuous distraction report a similar high percentage of neurological complications. The most serious complication in our series was a pulmonary embolism in a 32-year-old woman. Apart from the ankle arthroscopy, she underwent open surgery during the same anaesthesia, requiring 6 weeks of cast immobilization. The combination of cast immobilization, her use of oral contraception and smoking (a package a day) may have made her more prone to the development of embolism. She did not receive prophylactic anticoagulants.

In order to find an explanation for the difference in complication rate in our series (3.5 %) and those reported in other series (average 10.3 %), we analysed to following aspects: definition of complications, type of complication registration, type of surgical procedures, surgical experience or technique of the procedure. Concerning definition of complications, we used the following definition: ‘every event that arises as an additional problem during or following the procedure and is secondary to it’. When we compare the type of our complications to those reported in literature, there is no difference [2, 14, 18, 26, 37]. We feel that an underestimation of complications did not occur since we used a prospective national registration system. Apart from that, we checked all patient charts. Concerning the type of operative procedures, we have listed our indications. They do not differ from other authors [2, 11, 14, 17, 18, 26, 27, 37]. Concerning surgical experience, in our series 33 surgeons were involved. Other studies often include arthroscopic procedures of only one experienced orthopaedic surgeon. Our low complication rate in spite of the involvement of a large number of low volume surgeons is therefore remarkable and in favour of the procedure.

**Fig. 2:**
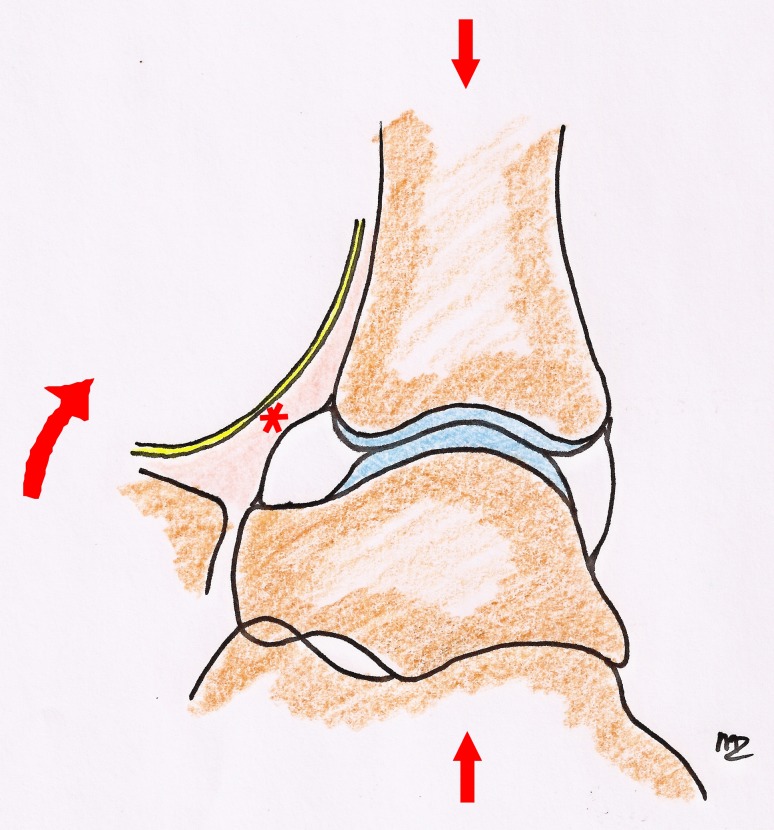
Dorsiflexed ankle without distraction. The *asterisk* indicates that the nerves and vessels are relaxed in a protective subcutaneous layer and with a considerable anterior working space in front of them. They are therefore less prone to be injured

**Fig. 3:**
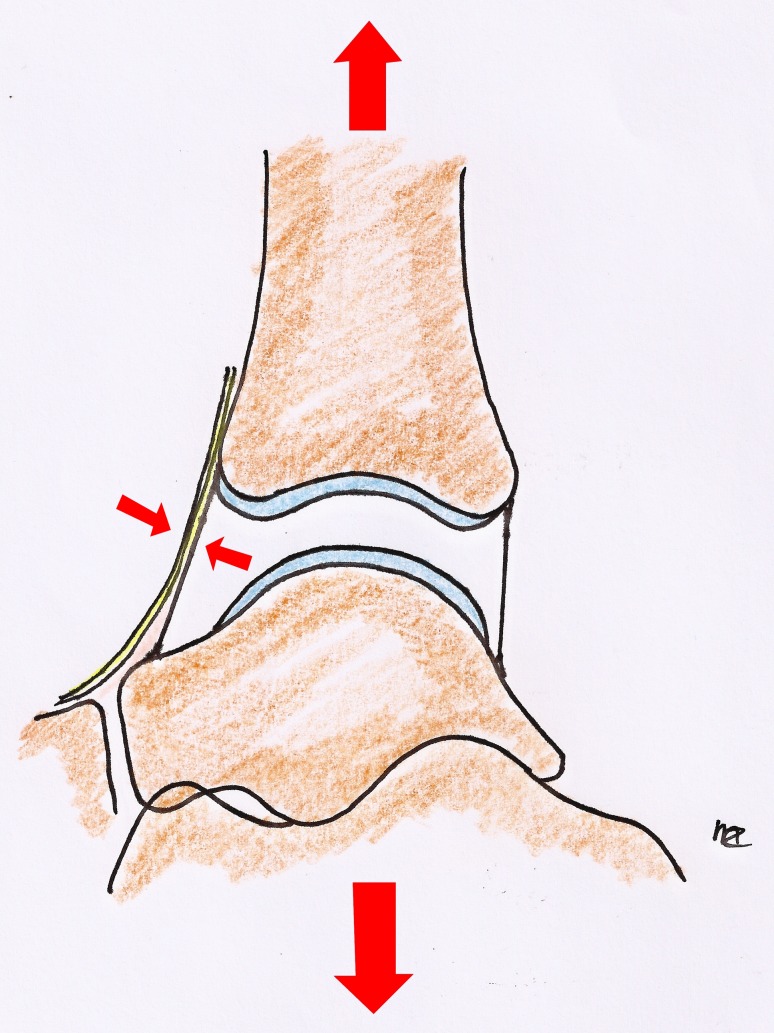
Distracted ankle. The small *arrows* point at the nerves and vessels that are under tension in this position. In a distracted ankle, they are more vulnerable to iatrogenic injury

We conclude that the dorsiflexion technique that we use to perform the arthroscopic procedure is the main reason for the low percentage of complications when compared to other series using a continuous distraction system. Some details and technical conditions of our approach are important to mention. Like others we identify the superficial peroneal nerve by plantarflexing and inverting the foot and marking it on the skin [13, 34]. It was shown by De Leeuw et al. [9] that the superficial peroneal nerve moves in a lateral direction in relation to the skin when the ankle is moved back from this inverted position to the neutral position. This movement is mean 3.6 mm to the lateral side. It is therefore important to stay on the medial side of the marking. We enter the joint by blunt dissection with the joint in dorsiflexion. In this dorsiflexed position, the nerves and vessels are relaxed and can thus move away when a blunt instrument is introduced (Fig. 2). When compared to the distracted and slightly plantarflexed position, nerves and vessels are stretched and cannot move, making them more vulnerable to iatrogenic damage (Fig. 3). In a recent series concerning ankle arthroscopy, foot distraction straps and continuous application of distraction were used during portal creation and throughout the procedure. The continuous distraction was held responsible for the high percentage (5.4 %) of neurological complications [45]. Our percentage of neurological complications of 1.9 % compares favourably to the average of 3.7 % reported in the literature [2, 3, 11, 14, 17, 18, 26, 27, 37]. We believe that this technique of portal creation and introduction and changing of instruments in a dorsiflexed position without joint distraction is the main reason for the low percentage of nerve complications in our series. Another important aspect of working in the anterior joint area is the distance between the anterior joint space and the overlying neurovascular structures. It has been recently shown that the distance between the anterior tibial rim and the neurovascular structures is significantly reduced with the ankle in the distracted position [8]. The neutral or dorsiflexed position without distraction increases the anterior safe working area [8].

Several authors mention the occurrence of a false aneurysm or other vascular lesions [5, 7, 20–23, 25, 28, 30, 31, 43]. Although the anatomical course of the anterior tibial artery is known, medial deviation occurs in approximately 3.5 % and lateral deviation in 5.5 % of patients in anatomical studies [42, 44]. In an anatomical analysis, it was found that in 6.2 % the anterior tibial artery was located near the anterolateral portal [33]. Vessels are easily damaged when they do not follow the best-known anatomical route [12]. It is important to always pre-operatively palpate the pulsations to detect where arteries are located. Working in dorsiflexion helps to prevent vascular complications not only because it allows for a larger safe working area, but the vessels are also less likely to be damaged since they can move aside when accidently touched by a blunt instrument.

**Fig. 4:**
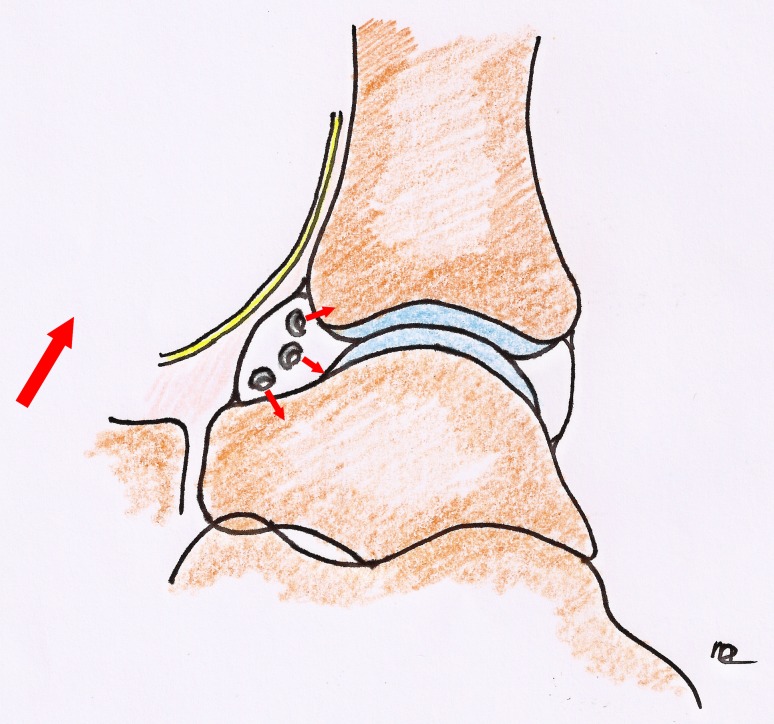
Opening of shaver. In the anterior working space of the dorsiflexed ankle, the opening of the shaver should always point towards the joint. This prevents iatrogenic damage to neurovascular structures

Another aspect in the prevention of complications is the position of the shaver. In the anterior working area, the opening of the shaver should be directed towards the ankle joint, and not towards the skin (Fig. 4). In a dorsiflexed, undistracted position, this is easily accomplished, because relaxation of the anterior structures of the ankle allows for a considerable working space. In a distracted ankle, however, this space is limited [8].

Looking specifically at complications in hindfoot endoscopy, we found a rate of 2.3 %. This is lower than what was found in a recent study, specifically on this subject, by Nickisch et al. [27]. One explanation may be that in 31.7 % a distraction technique was used in which a tensioned wire was placed transversely through the calcaneus. However, separate rates were not described. Also for hindfoot endoscopy, we recommend intermittent non-invasive distraction to prevent tension on neurovascular structures.

In this series there were 2 patients with a deep infection (0.15 %). In one patient this occurred 12 days after surgery following sinus tract formation, which was not treated with prophylactic antibiotics. The other occurred a few days after surgery. Our percentage of infection compares favourably to the average 0.6 % infection rate in other series (Table 1).

Ferkel et al. [13] have summarized some aspects in prevention of infection after ankle arthroscopy. These include immobilization of the ankle joint the first week post-operatively and the administration of prophylactic antibiotics. We do not believe that these two factors play an important role. On the contrary, we do not immobilize our patients post-operatively but stimulate the patient to move the ankle in active dorsiflexion a few times an hour starting on the day of surgery. One of the great benefits from the minimally invasive surgery over open surgery is this early mobilization. We tolerate patients to mobilize with partial weight bearing on the day of surgery. Patients are stimulated to use their crutches for 4–5 days maximum. Neither do we give prophylactic antibiotics. Our low infection rate seems to justify our protocol.

We agree with Ferkel et al. that it is important to suture the portals, and we do this in all patients. The most important precaution to prevent infection, as well as to prevent sinus tract formation, however, is probably to take care not to remove the subcutaneous tissue at the portal site. In a dorsiflexed position the anterior working area opens up when fluid is brought into the joint. The skin and subcutaneous tissues thus move away from the joint. In this position it is possible to perform the operative procedure without removing subcutaneous tissue. In a distracted, slightly plantarflexed position the anterior working area disappears. The skin and stretched subcutaneous layers move towards the joint [8], making them more vulnerable to iatrogenic removal. Removal of subcutaneous fatty tissue at the portal site creates an easy entry point for bacteria. Limited tourniquet time is another important factor. Before surgery the patient should have a proper diagnosis. Current additional diagnostics like MRI or CT scan make it possible to achieve this. A treatment plan must have been made pre-operatively. Diagnostic arthroscopy preceding an arthroscopic intervention unnecessarily lengthens the procedure. We therefore feel that diagnostic arthroscopy is obsolete.

The application of traction has been mentioned earlier as a possible cause of complications [45]. Concerning distraction, we can identify three options: invasive skeletal distraction, continuous soft tissue distraction or intermittent soft tissue distraction [29, 39]. Invasive skeletal distraction has been associated with a number of specific complications like fracture of the fibula, stress fracture of the tibia, pin breakage, vascular damage, ligament damage, pintrack drainage and pintrack infection [14, 34]. Neurological complications following skeletal distraction and continuous soft tissue distraction have been described by several authors [1, 14, 19, 37].

Soft tissue distraction increases the risk of complications because it creates tension on neurovascular structures and gives direct pressure on the skin. Dorsiflexion without distraction provides relaxed structures that are subsequently less prone to be injured. Tension on nerves and vessels should be avoided when the incision is made. Portals should therefore be created while the ankle is in a neutral or dorsiflexed position. If during the procedure distraction is desired to facilitate working in the posterior joint compartment, it can be accomplished by using a resterilizable non-invasive ankle distraction device [39]. The main advantage of the device is that the choice to perform distraction can be made at any time during the arthroscopic procedure. Applying the distraction only when indicated limits the overall distraction time. In our series of patients, none of the complications could be related to the use of this intermittent soft tissue distraction.

For most operative procedures, there is a learning curve. Consequently, a higher percentage of complications can be expected during the initial procedures. We could not demonstrate a learning curve in our series, however. For the surgeons in this study that performed more than 20 procedures, the complication rate was indeed lower when compared to the low volume surgeons. It was 5.7 % for their first 10 procedures, versus 3.5 % overall. However, the difference was not statistically significant. Most likely, there is a learning curve, but we could not demonstrate this because of the low number of complications.

We analysed whether we could identify intrinsic factors like demographics or type of procedure that influence the percentage of complications. Although there were differences in complication rates between the indication groups, no significant difference could be demonstrated between them, which could be due to the low overall rate of complications. The only variable of significant influence on the complication rate was age of the patient.

The present study has some limitations, concerning study design and possible under-registration of complications. Our study design did not allow us to compare the complication rate of different surgical techniques within our study. Since we only use one well-established technique in our hospital, we cannot compare complication rates for different surgical techniques within our study. This may lead to bias. Furthermore, we failed to identify statistically significant risk factors, accept for age and the effect of a learning curve. It is very well possible that some risk factors did not reach significance because a larger group of patients is needed. Also, our patient population was relatively heterogenous, having undergone a variety of procedures for several different diagnoses. Finally, certain complications like iatrogenous cartilage damage are difficult to examine and therefore not scored. This will have led to an under-registration of complications. However, this complication is not scored in other studies either, and complication rates in published literature can still be compared.

The current study is relevant because it shows a low complication rate related to a specific arthroscopy technique. Today, many different techniques are used to perform anterior and posterior ankle arthroscopy. One should always aim for the lowest possible number of complications. Use of a meticulous technique can aid in reaching this goal.

## Conclusion

We conclude that anterior ankle arthroscopy by means of a dorsiflexion approach and a 2-portal hindfoot approach for posterior ankle arthroscopy lead to low complication rate. This fact points towards the benefits of working with the dorsiflexion method and with the use of intermittent soft tissue distraction. The overall percentage of complications for hindfoot endoscopy compares favourably to anterior ankle arthroscopy (2.3 vs 3.5 %).
